# Molecular characterization and antimicrobial-resistance gene profile of *Staphylococcus aureus* strains isolated from ovine mastitis in Jordan

**DOI:** 10.14202/vetworld.2025.270-279

**Published:** 2025-02-13

**Authors:** Mohammad H. Gharaibeh, Tasneem A. Mahafzah, Luay F. Abu-Qatouseh, Malak Khanfar, Amir Abdulmawjood

**Affiliations:** 1 Department of Basic Veterinary Medical Science, Faculty of Veterinary Medicine, Jordan University of Science and Technology, P. O. Box 3030 Irbid, 22110, Jordan; 2 Department of Pharmacology and Biomedical Sciences, Faculty of Pharmacy, University of Petra, Amman, Jordan; 3 Department of Biotechnology and Genetic Engineering, Faculty of Science and Arts, Jordan University of Science and Technology, P. O. Box 3030 Irbid, 22110, Jordan; 4 Institute of Food Quality and Food Safety, Research Center for Emerging Infections and Zoonoses (RIZ), University of Veterinary Medicine Hannover, Foundation, Bünteweg 17, 30559 Hannover, Germany

**Keywords:** antimicrobial resistance, ovine mastitis, PFGE, spa typing, *Staphylococcus aureus*, zoonotic transmission

## Abstract

**Background and Aim:**

Ovine mastitis, particularly subclinical mastitis caused by *Staphylococcus aureus*, poses significant economic and health challenges in livestock management. This study aimed to investigate the molecular characteristics and antimicrobial-resistance gene profiles of *S. aureus* isolated from subclinical mastitis in northern Jordan and assess the zoonotic potential of these isolates.

**Materials and Methods:**

A total of 283 milk samples from ovines and 24 nasal swabs from animal handlers across three northern Jordanian governorates were analyzed. Bacterial isolates were identified phenotypically and genotypically, with antimicrobial susceptibility tested using disk diffusion and minimum inhibitory concentration assays. The presence of antimicrobial-resistance genes (ARGs) was analyzed through polymerase chain reaction, and genetic relatedness was determined using pulse-field gel electrophoresis (PFGE) and *spa* typing.

**Results:**

*S. aureus* was detected in 16 (6%) ovine milk samples and four nasal swab samples from animal handlers. High resistance rates were observed for penicillin G, oxacillin, and cefoxitin (25% each). ARGs, including *mecA*, *blaZ*, *aph(3′)-III*, and *ant(4′)-Ia*, were detected in 25% of isolates. PFGE revealed a high genetic similarity among isolates, while *spa* typing identified three types, with *t1534* predominating (81.25%). Limited cross-transmission between ovines and handlers was noted.

**Conclusion:**

The study highlights *spa* type *t1534* as the dominant genotype in ovine mastitis in Jordan and underscores the low zoonotic transmission risk from ovines to animal handlers. The findings emphasize the importance of antimicrobial stewardship and enhanced mastitis management strategies.

## INTRODUCTION

Mastitis is one of the most common diseases in sheep, and it is characterized by inflammation of the mammary gland [1]. Mastitis can cause a significant decrease in milk production and a change in milk’s physical and chemical properties, resulting in great economic losses in the dairy industry and animal welfare worldwide [2]. *Staphylococcus aureus* is a common commensal organism in many mammalian species [3]. However, under appropriate circumstances, *S. aureus* can become an opportunistic pathogen, causing mild-to-severe infections such as mastitis [4]. Therefore, *S. aureus* is one of the most common pathogens responsible for clinical and subclinical ovine mastitis in Jordan and worldwide [1, 5]. This is the first study in Jordan to investigate the use of *spa* typing and pulse-field gel electrophoresis (PFGE) for the typing and detection of resistance genes of *S. aureus* in ovine. Molecular classification techniques, such as PFGE and spa classification, significantly enhance the genetic diversity and transmission dynamics of *S. aureus*. In livestock and human populations, these techniques are combined to improve the tracking of antibiotic-resistant strains [6, 7]. This study examined the molecular characteristics of *S. aureus* in ovine mastitis and assessed the zoonotic potential of the resistant strains, especially their ability to transmit to animal handlers. This research addresses antimicrobial resistance, an urgent public health challenge, by adopting a One Health approach focusing on genetic relatedness between ovine and human isolates [8]. However, few studies have been conducted in Jordan to determine the prevalence of *S. aureus* mastitis in Awassi sheep with clinical and subclinical mastitis. The cases were diagnosed using a somatic cell count and the California mastitis test (CMT) [9–11]. The milk sampling population ranged from 46 to 283 milk samples, while the prevalence of *S. aureus* ranged from 6% to 36% in different geographical regions from the north to the south of the country [9–11]. Antibiotic resistance has become a global concern for *S. aureus*, especially in the context of zoonoses, where transmission occurs between animals and humans. As a result, multidrug-resistant (MDR) strains are more likely to occur in areas where antibiotics are widely used in agricultural and veterinary settings. Methicillin-resistant *S. aureus* (MRSA) isolates, encoded by *mecA* gene, complicate treatment strategies for mastitis in small ruminants [12]. The use of antibiotics in veterinary and agricultural practices, as well as their misuse, contributes to the development of AMR and the dissemination of MDR bacteria [13]. Antimicrobial resistance increases treatment costs and animal mortality, threatening the dairy and livestock industries [14]. *S. aureus* develops antimicrobial resistance through several mechanisms, including horizontal gene transfer, genetic alteration, efflux pump function, and biofilm formation. The molecular typing of *S. aureus* isolates is essential for identifying the dominant genotypes and establishing genetic relatedness [5]. However, limited studies have been conducted in Jordan to investigate *S. aureus* isolates from ovine mastitis. It is anticipated that the identification of *S. aureus-*resistant genes and the identification of molecular types will enhance our mastitis control strategies. Typing methods such as PFGE and *spa* typing have excellent discriminatory power and have been successfully employed to identify *S. aureus* clonality responsible for outbreaks around the world. In addition, they have been used to study patterns of strain dissemination [15].

Therefore, this study aimed to molecularly characterize *S. aureus* strains isolated from ovine mastitis and evaluate their antimicrobial-resistance profiles. PFGE and *spa* typing methods were utilized to determine genetic relatedness. Further, it emphasizes that animal handlers in Jordan may also be infected. These findings will help develop antimicrobial stewardship policies in the livestock sector and improve mastitis control strategies.

## MATERIALS AND METHODS

### Ethical approval

The study protocol was approved by the Animal Care and Use Committee of Jordan University of Science and Technology (JUST) Animal Care and Use Committee (JUST – ACUC approval number 16.3.3.56). Nasal samples were collected by a trained person as per the standard sample collection procedure.

### Study area and sample collection

This cross-sectional study was conducted between March 2018 and September 2018. A total of 283 milk samples were collected from suspected cases of subclinical ovine mastitis. Milk samples were collected from ovine raised in three major governorates in northern Jordan: Irbid, Mafraq, and Jerash. Milk samples were selected based on availability, with suspected cases of subclinical ovine mastitis as the criterion for inclusion. CMT was used to identify subclinical mastitis in 283 milk samples. Approximately 10 mL of quarter milk samples were collected from selected sheep using an aseptic technique, placed in an ice box, and transported to the laboratory within 4–6 h. In addition, 24 nasal swab samples were collected from animal handlers stationed at ovine farms. Swabs were transported and processed as described by Alekish *et al*. [16]. The samples were processed at the Laboratory of Microbiology at the Faculty of Veterinary Medicine, Jordan University of Science and Technology.

### *S. aureus* isolation and identification

To isolate *S. aureus*, 10 µL loopful of the milk sample was used to inoculate nutrient agar, 5% sheep blood agar, and mannitol salt agar (MSA) (Oxoid, UK) and incubated at 37°C for 24–48 h. Colonies that exhibited clear β-hemolysis on blood agar and those that appeared yellow with a surrounding yellow zone on MSA agar were reported as suspected *S. aureus* colonies. The suspected colonies were further confirmed using catalase and coagulase biochemical tests with the Mastastaph Kit (Mast Group, UK). Catalase- and coagulase-positive isolates were preserved in Mueller–Hinton broth (MHB) (Oxoid, UK) containing 30% glycerol for further identification. For the molecular confirmation of *S. aureus* isolates, DNA was extracted and purified using a commercial kit (Qiagen, Germany) according to the manufacturer’s instructions, with lysozyme and lysostaphin pre-incubation steps (10 mg/mL and 10 g/mL, respectively) for 45 min. All isolates were confirmed to be *S. aureus* by amplifying the thermonuclease gene (*nuc* gene), as described by Alekish *et al*. [16]. *S. aureus* isolates recovered from ovine milk samples were coded as (O), whereas isolates recovered from human nasal swabs were coded as (H). Polymerase chain reaction (PCR) was performed using the primer sequences and conditions listed in (Table 1) [2, 15–19].

**Table 1:** Primers sequence, annealing temperature, and amplicon size of *Staphylococcus aureus* species specific and antimicrobial resistance genes.

Target gene	Primer pair	Sequence (5′→3′)	Annealing temperature	Amplicon size (bp)	Reference
*TetK*	F R	5’- TATTTTGGCTTTGTATTCTTTCAT-3’ 5’- GCTATACCTGTTCCCTCTGATAA-3’	55°C	360	[2]
*TetM*	F R	5’- AGT TTT AGC TCA TGT TGATG 5’- TCCGACTAT TTAGACGAC GG-3’	55°C	158	
*Nuc*	F R	5’- GCGATTGATGGTGATACGGTI-3’ 5’ AGCCAAGCCTTGACGAACTAAAG C-3’	53°C	267	[15]
*aac (6)/aph (2)*	F R	5’- GAA GTA CGC AGA AGA GA 5’- ACATGGCAAGCT CTA GGA-3’	55°C	491	[16]
*aph (3′)-III*	F R	5’- AAATACCGCTGCGTA-3’ 5’- CATACTCTTCCGAGCAA-3’	58°C	242	
*ant (4)-Ia*	F R	5’CAAACTGCTAAATCGGTAGAAGCC-3’ 5’GGAAAGTTGACCAGATTACGAACT-3’	58°C	294	
*MecA*	F R	5’- CGGTAACATTGATCGCAACGTTC-3’ 5’CTTTGGAACGATGCCTAATCTCAT-3’	53°C	162	[17]
*BlaZ*	F R	5’- GCTTGACCACTTTTATCAGC-3’ 5’- GCTTGACCACTTTTATCAGC-3’	50°C	517	[18]
*ErmA*	F R	5’- ATCGGATCAGGAAAAGGACA-3’ 5’- CACGATATTCACGTTTTACCC-3’	55°C	190	
*ErmB*	F R	5’- AAGGGCATTTAACGACG AAA-3 5’- CTGTGGTATGGCGGGTAAGT-3’	55°C	423	
*ErmC*	F R	5’- TGAAATCGGCTCAGGAAAAG- 5’- CAAACCCGTATTCCACGATT-3’	55°C	299	
*Str*	F R	5’GAGGGTTCAAGAACTAATG3’ 5’AACACCCTTTGCTACATAC-3’	58°C	432	[19]

### Antimicrobial susceptibility test

#### Standard disk diffusion (DD) method

All confirmed *S. aureus* isolates were tested for susceptibility against a panel of 12 antimicrobial agents most commonly used in the literature for the treatment of clinical mastitis cases and *S. aureus* infection in humans using the standard Kirby–Bauer DD procedure. After overnight culture on Mueller–Hinton Agar (Oxoid, UK), three to five colonies were suspended in MHB (Oxoid), and the bacterial suspension was adjusted to 0.5 McFarland (0.132 OD) using a SmartSpec 3,000 spectrophotometer (Bio-Rad Laboratories, UK) at 600 nm. The bacterial suspension (0.5 McFarland 10^5^ cfu/mL) was spread using a sterile cotton swab on the Mueller–Hinton plate (Oxoid). Within 10 min, at room temperature (25°C), the disks were applied on the surface of the medium. The plates were incubated at 37°C for 18–24 h, the zone of inhibition around the disk was measured in millimeters according to Gharaibeh *et al*. [19] and following the Clinical Laboratory Standard Institute (CLSI) guidelines, 2017 [17]. The panel includes penicillinase-stable penicillin G: (50 µg) penicillin G (P), (30 µg) oxacillin (OX), (10 µg) ampicillin (AMP); (10 µg) cefoxitin (Fox), aminoglycosides: (10 µg) gentamycin, (30 µg) kanamycin (K), 10 µg streptomycin (STR); macrolides: (15 µg) erythromycin (E); tetracyclines (TET): (30 µg) TET; lincosamides: (2 µg) clindamycin (CN); and folate pathway inhibitors: (5 µg) trimethoprim-sulfamethoxazole (SXT), and (10 µg) fusidic acid (FUS) [19]. The inhibition zones around each disk were measured, and the isolates were classified as either resistant, intermediate, or susceptible to each antimicrobial agent based on the criteria and breakpoints mentioned in the 2017 CLSI guidelines [17].

### Minimum inhibitory concentration (MIC) test

MICs were determined for five antimicrobial agents – P, STR, E, K, and oxytetracycline (OTC) – using the standard broth microdilution procedure described in the 2017 CLSI guidelines [17]. In brief, antibiotic stock solutions were prepared and serially diluted in a 96-well polystyrene microtiter plate to achieve a final concentration gradient between 0.0625 µg/mL and 64 µg/mL in each lane. Bacterial suspensions from fresh overnight cultures were diluted by a factor of 1:100 in sterile MHB to achieve McFarland turbidity of 0.5. For each well, 50 µL of suspension was added to 50 µL of diluted antibiotic, resulting in a final volume of 100 µL; the plates were incubated for 16–20 h at 35°C. The wells of the 12^th^ column were filled with sterile MHB for sterility testing. Using transmitted light, the wells were monitored for visible growth after incubation. Based on the MIC breakpoints described in the 2017 CLSI guideline, the isolates were classified as resistant, susceptible, or intermediately resistant [17].

### Detection of antimicrobial-resistance genes (ARGs) using PCR

To detect 11 ARGs, 11 uniplex PCR amplification reactions were performed. These genes encode resistance to beta-lactams (*blaZ*, and *mecA*), aminoglycosides (*aac(6′)/aph(2′′)*, *aph(3′)-III*, *ant(4′)-Ia*, and *str*), macrolides (*ermA*, *ermB*, and *ermC*), and TET (*tetM* and *tetK*). Reactions were performed using primer sequences and PCR conditions as previously described [18, 20–24]. The amplification of DNA was performed on a single-plex platform for all genes. The standard PCRs were performed as follows: Total volume 25 µL that contained 4 µL 5× Hot Fire pol Blend master mix (Solis Biodyne, Estonia), 1 µL of each primer, 2 µL template DNA, and 18 µL nuclease-free water (NFW). These reactions were performed using a Thermal Cycler System (Bio-Rad) with the following PCR conditions: pre-incubation at 94°C for 15 min, 37 cycles (pre-denaturation at 94°C for 1 min, annealing at different temperatures for 30 s, extension at 72°C for 1 min), and final extension step at 72°C for 5 min. The amplified DNA fragments were separated on a 1.5% agarose gel using 10 µL of the PCR product stained with ethidium bromide. Finally, the amplified DNA fragments were visualized using the documentation system Endurotmgds (Labnet, PCR). PCR was conducted using the primer sequences and conditions listed in (Table 1) [2, 15–19].

### Molecular typing of *S. aureus* isolates

#### PFGE

PFGE of all *S. aureus* isolates was performed as described by Alekish *et al*. [16]. Briefly, to digest bacterial DNA, New England Biolabs’ *SmaI* endonuclease enzyme (New England Biolabs, USA) was applied for 18 h at 37°C. After electrophoresis was completed, a linear ramping factor of 120u and 5–50 pulses were applied at 14°C and 6 V/cm using an electrophoresis device (Bio-Rad). A standard pattern was included in the gels to facilitate the comparison of the digitally normalized PFGE profiles (Lambda Ladder PFG Marker, New England Biolabs). Computer software (PyElph 1.4, Informer Technologies, Inc., USA) was used to obtain and analyze images of the gels. Clusters were determined using dice coefficients represented by unweighted pair groups based on the arithmetic averages (UPGMA) clustering method with 1% band position tolerance and 0.5% optimization settings. To estimate the significant genetic variation/similarity between bacterial isolates, Python software (http://sourceforge.net/projects/pyelph) was employed to construct a comprehensive.

#### spa typing

*Spa* typing was performed for all *S. aureus* isolates by amplifying the hypervariable X-region of the *spa* gene, as described by Shopsin *et al*. [25]. Briefly, the primer sequences for the *spa* gene forward and reverse primers of *spa*-1113f: 5′ TAA AGA CGA TCC TT C GGT GAG C3′ and *spa*-1514r: 5′ CAG CAG TAG TGC CGT TTG CTT 3′, respectively. PCR consisted of a 2-µL aliquot of extracted DNA added to 10 µL of 5× PCR master mix FirePol^®^ (Solis BioDyne), 36 µL of NFW, and 1 µL of 10 pmol/mL from each of the forward and reverse primer pairs of the *spa* gene to achieve a final volume of 50 µL. The amplification conditions included an initial 10 min at 95°C; 30 cycles of 30 s at 95°C, 30 s at 60°C, and 45 s at 72°C; and a final extension at 72°C for 10 min. The *spa* gene sequence analysis was conducted using Ridom StaphType software (Ridom GmbH, Würzburg, Germany) (www.spaserver.ridom.de).

### Statistical analysis

Data were analyzed using SPSS version 25 (IBM Corp., NY, USA). Descriptive statistics were used to summarize the data, including the prevalence of *Staphylococcus aureus* isolates, antimicrobial-resistance profiles, and the distribution of ARGs. The Chi-square test was employed to examine associations between categorical variables, such as the prevalence of *S. aureus* in different geographic regions and among sample sources (ovine milk vs. human nasal swabs).

Phylogenetic relationships between *S. aureus* isolates were assessed using Dice coefficients and UPGMA clustering analysis for PFGE data. Genetic similarity thresholds were set at ≥80% to define closely related clusters. *spa* typing diversity was evaluated by the relative frequency of detected types.

The MICs of antimicrobial agents were analyzed to determine resistance profiles. Resistance rates were compared between ovine and human isolates using appropriate non-parametric tests due to the small sample size.

All tests were two-tailed, and a p-value ≤ 0.05 was considered statistically significant. Results are presented as frequencies, percentages, and significance levels where applicable.

## RESULTS

### Isolation and identification of *S. aureus*

A total of 283 subclinical mastitis samples were collected from 16 ovine-raising farms in Irbid (22.3%, n = 63), Al-Mafraq (42.4%, n = 120), and Jerash (35.5%, n = 100) (Table 2). Using the culture detection method, it was found that 49% of subclinical milk samples tested positive on blood agar and MSA, but only 5.7% (n = 16) were molecularly confirmed as *S. aureus* using PCR for the *nuc* gene (Table 2). Moreover, 16% (n = 4) of the isolates were recovered from animal handlers; three isolates were recovered from farms in Al-Mafraq, while the fourth isolate was recovered from Jerash. In addition, in Irbid, the proportion of positive cultures was significantly higher (p ≥ 0.05) compared with Jerash. The proportion of positive culture was significantly higher (p ≥ 0.05) in animal handlers than in ovine milk (Table 2).

**Table 2:** Prevalence of *Staphylococcus aureus* in ovine milk and animal handlers from Jordan.

Area/source	Farms (n)	Number of samples (%)	Number of positive cultures (%)*	Number of *Staphylococcus aureus* isolates of positive culture (%)
Irbid				
Ovine	3	63 (22.3)	39 (62)a	2 (5.1)
Human	1	6 (8.3)	6 (100)	1 (16.6)
Mafraq				
Ovine	8	120 (42.4)	57 (48)	9 (15.8)
Human	6	13 (54)	11 (84.6)	2 (18.1)
Jerash				
Ovine	3	100 (35.3)	44 (44)a	5 (11.4)
Human	2	5 (20.8)	3 (60)	1 (20)
Total				
Ovine	14	283 (100)	140 (49)b	16 (11.4)
Human	9	24 (100)	19 (79)b	4 (16.6)

* Same letters in the column are statistically significant at p *≤* 0.05. ^a^: indicates statistically significant differences among ovine samples from different regions. ^b^: indicates statistically significant differences between ovine and human samples at p *≤* 0.05

### Antimicrobial sensitivity testing

The antimicrobial susceptibility profiles of *S. aureus* isolates from ovine and animal handlers are listed in Table 3 and were established using the Kirby–Bauer DD method. Based on the resistance profiles of all isolates and following the definition of acquired resistance proposed by Magiorakos *et al*. [26], 12.5% of the isolates were classified as MDR. The highest resistance rates were observed against P (25%, n = 4), followed by OX (18.8%, n = 3), TET (18.8%, n = 3), and STR (18.8%, n = 3) of the isolates demonstrating resistance to each of the four antimicrobial agents. Moreover, moderate rates of resistance were observed against AMP (12.5%, n = 2) and FOX (12.5%, n = 2), whereas 6.25% (n = 1) of the isolates were resistant to E and K. All isolates were susceptible to SXT, GEN, CN, and FUS. In contrast, among the animal handler *S. aureus* isolates, P, and STR exhibited the highest resistance rates, with 100% (n = 4) of isolates. In addition, there wasa significant level of resistance to OX (75%, n = 3), AMP (75%, n = 3), and FOX (75%, n = 3). Moderate resistance to E and CN was observed (25%, n = 1) of isolates. However, all isolates were susceptible to TET, K, SXT, GEN, and FUS. The MIC values of P, STR, E, K, and OTC for each *S. aureus* isolate were recorded (Table 4). The highest resistance rate was observed for penicillin (25%, n = 4), followed by kanamycin (18.7%, n = 3) and oxytetracycline (13%, n = 2). All isolates were susceptible to STR and erythromycin.

**Table 3:** Antimicrobial resistance profiles of *Staphylococcus aureus* isolated from subclinical mastitis milk.

Antimicrobials	Susceptible (S) and resistant (R) isolates			

	Ovine *Staphylococcus aureus* (n = 16)		Human *Staphylococcus aureus* (n = 9)	
	
	S No. (%)	R No. (%)	S No. (%)	R No. (%)
Penicillin	12 (75%)	4 (25%)	0 (0%)	4 (100%)
Oxacillin	13 (81.2)	3 (18.8)	1 (25)	3 (75)
Tetracycline	13 (81.2)	3 (18.8)	4 (100)	0 (0)
Streptomycin	13 (81.2)	3 (18.8)	1 (25)	4 (100)
Ampicillin	14 (87.5)	2 (12.5)	1 (25)	3 (75)
Cefoxitin	14 (87.5)	2 (12.5)	1 (25)	3 (75)
Erythromycin	15 (92.7)	1 (6.3)	3 (75)	1 (25)
Kanamycin	15 (92.7)	1 (6.3)	4 (100)	0 (0)
Sulphamethoxazole/trimethoprim,	16 (100)	0 (0)	4 (100)	0 (0)
Gentamicin	16 (100)	0 (0)	4 (100)	0 (0)
Clindamycin	16 (100)	0 (0)	3 (75)	1 (25)
Fusidic acid	16 (100)	0 (0)	4 (100)	0 (0)

**Table 4:** Resistance profiles of *Staphylococcus aureus* isolates recovered from ovine milk.

Isolates ID	MIC (μg/μL)					

	Penicillin		Kanamycin		Oxytetracycline	
O7	64	R	8	S	4	S
O1	64	R	1	S	128	R
O16	0.125	S	16	S	4	S
O5	0.0625	S	16	S	2	S
O8	1	R	16	S	4	S
O6	1	R	2	S	128	R
O9	1	R	4	S	2	S
O10	1	R	32	I	2	S
O11	0.0625	S	4	S	2	S
O12	0.0625	S	4	S	2	S
O15	1	R	8	S	1	S
O4	64	R	16	I	4	S
O3	16	R	16	I	1	S
O13	0.125	S	8	S	1	S
O14	0.25	R	4	S	4	S
O2	0.0625	S	8	S	2	S

MIC=Minimum inhibitory concentration, S=Susceptible, I=Intermediate, and R=Resistant

### Antibiotic resistance genes

**Figure 1:**
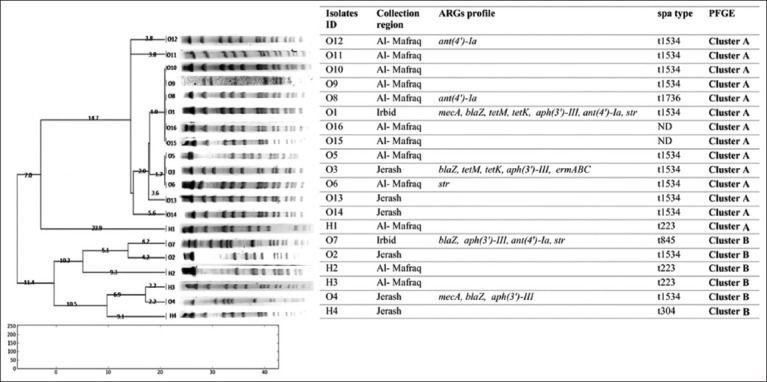
Dendrogram of *SmaI*-digested DNA patterns from *Staphylococcus aureus* isolates from sheep mastitis.

A total of 10 of the 11 ARGs investigated in this study were found in the isolates, as shown in (Figure 1). The *blaZ*, *aph(3′)-III*, and *ant(4′)-Ia* genes were the most detected ARGs in the ovine *S. aureus* isolates, with a prevalence of 25% (n = 4), followed by the aminoglycoside-resistance gene *str* (18.75%, n = 3) and *mecA* gene (12.5%, n = 2) encoding methicillin resistance. Moreover, the macrolide-resistance genes (*ermABC*) were detected in only 1 (6.25%) isolate. Based on the detection of *mecA* gene, 12.5% (n = 2) of the isolates were reported as MRSA, and the isolates were recovered from Irbid and Jerash.

## PFGE

*S. aureus* isolates were analyzed using PFGE to determine the relatedness of isolates obtained from different sources (Figure 1). The analyzed samples were categorized into two main clusters. Cluster A comprises four related groups with a similarity index >85%. Cluster B consisted of three groups with a similarity index of approximately 90%. According to PFGE, isolates O1, O8-O10, O15, and O16 were identical and more closely related to isolates O3, O5, and O6 in cluster A. In addition, O3 and O6 were highly related to O13 with a 96% similarity. Alternatively, cluster B contained isolates that were less genetically related to those assigned to O2, O4, and O7. On the other hand, human *S. aureus* isolates (H2-H4) belong to cluster B, whereas H1 belongs to cluster A with less relationship to ovine isolates.

### *spa* typing

**Figure 2:**
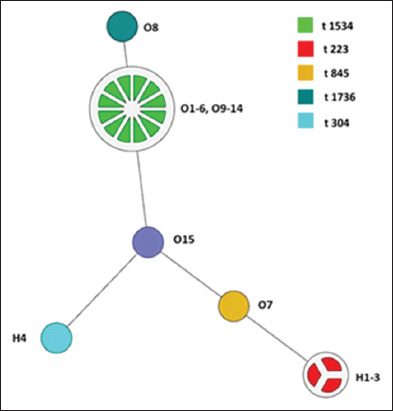
Dendrogram of *Staphylococcus aureus* ovine subclinical mastitis isolates discriminated by *spa* typing.

According to this study, 14/16 ovine sheep samples were successfully classified as *spa* types. *S. aureus* isolates exhibited three distinct *spa* types associated with ovine strains of *S. aureus* that cause subclinical ovine mastitis (Figures 1 and 2). *spa* type t1534 was detected in most isolates (81.25%, n = 13), while *spa* types t845 and t1736 were detected in only one isolate each (6.25%, n = 1). Among the 16 isolates, two *spa*-type isolates could not be identified. Regarding human *S. aureus* isolates, three of the four isolates belong to *spa* type t223, whereas the fourth isolate belongs to *spa* type t304.

## DISCUSSION

Globally, ovine mastitis causes significant economic losses in the dairy industry. Moreover, *S. aureus* is a major causative agent of clinical and subclinical ovine mastitis in Jordan. Only a few studies have been conducted in Jordan to determine the prevalence of *S. aureus* mastitis. In dairy production, subclinical mastitis represents a significant challenge due to its adverse effects on milk quality and yield. Among the pathogens, *S. aureus* is the most common. In Jordan, the milk sampling population ranged from 46 to 283 milk samples. The prevalence of *S. aureus* ranges from 6% to 36% in different geographical regions, from the north to the south of the country [9–11]. Research conducted in Egypt identified *S. aureus* as a pathogen responsible for subclinical bovine mastitis, exhibiting various virulence factors [27]. Similarly, subclinical mastitis is highly prevalent in Europe, with *S. aureus* as the primary cause. A study in Germany found that herds had a high prevalence of *S. aureus* [28]. These findings emphasize the need for effective control measures and continuous monitoring to reduce the impact of subclinical mastitis on dairy production.

In this study, the occurrence of *S. aureus* isolates recovered from subclinical ovine milk samples was 6% (n = 16). Our findings are consistent with a previous study conducted by Lafi *et al*. [10] in the northern region of Jordan, which discovered a prevalence of 6.8%, compared with 4% in sheep and 8% in goats in a study by Bergonier *et al*. [29]. Nevertheless, additional research conducted in the middle and southern parts of Jordan revealed a higher incidence of *S. aureus* subclinical mastitis, ranging from 28% to 39% [11, 30]. The prevalence of *S. aureus* mastitis varies worldwide. Several factors may contribute to this, including the efficiency of farm management practices, climate, seasonality, and host factors, such as breed, age, and general udder health.

Among the antibiotics used in our study, *S. aureus* isolates were most resistant to penicillin G (25%), followed by oxacillin (18.8%), TET (18.8%), and STR (18.8%). Obaidat *et al*. [30] reported comparable resistance rates in Jordan. In another study by Mahlangu *et al*. [31] in Kenya, it was discovered that the extensive use of antimicrobial agents for treating ovine infections led to a significant rate of resistance to these drugs [30]. Furthermore, AMP (12.5%) and cefoxitin (12.5%) exhibited moderate levels of resistance, while erythromycin (6.25%) and kanamycin (6.25%) exhibited very low levels of resistance. These results are consistent with those previously reported in Jordan and Kenya [9, 31]. However, no resistance was observed to SXT-trimethoprim, gentamicin, CN, or FUS, all of which are no longer used in treating subclinical mastitis in sheep.

Several ARGs were identified in multiple strains, ranging from 6.25% for *ermA* to 25% for *blaZ*, *aph(3′)-III*, and *ant(4′)-Ia* genes (Figure 1). Gram-positive bacteria are the major cause of subclinical and clinical ovine mastitis. This condition is usually treated with systemic penicillin or occasionally with intramammalian aminoglycosides along with β-lactam antimicrobials or oxytetracycline. Penicillin has historically been considered an effective treatment for *S. aureus* infections; however, it did not take long for resistance to β-lactamases to develop (*blaZ* gene). In a previous study conducted in Brazil by França *et al*. [32], 25% of isolates contained the penicillin gene (*blaZ* gene) [32]. In this study, TET-resistant genes were detected in 12.5% of isolates from both *tetK* and *tetM*. Based on a previous study conducted in China, TET-resistant genes were detected in clinical bovine mastitis isolates, such as *tetK* (12.2%) and *tetM* (9.9%) [33].

Over the past five decades, macrolides have been used to treat infections caused by microorganisms that are classified as Gram-positive [7]. Several genes encode resistance proteins, including *ermC* and *ermA*. In this study, the macrolide-resistance gene showed resistance to *ermA* (6.25%), *ermC* (6.52%), and *Str* (19%). This result seems higher than those previously reported in Brazil [32]. Furthermore, *ermC* was found in China in 22.1% of bovine mastitis infections [33]. Another study conducted in Jordan identified *ermA* and *ermC* in 30%–40% of *S. aureus* isolates [5]. Aminoglycosides are bactericidal compounds used to treat infections caused by Gram-positive and Gram-negative bacteria. In this study, 25% (n = 4) of the isolates harbored aminoglycoside-resistant genes, including *aph (3′)-II* and *ant (4′)-Ia*. Our samples showed a higher prevalence of these genes than those previously reported in Sardinian mastitis [21]. In addition, *mecA* was detected in 12.5% of the isolates. This is particularly relevant because *mecA* encodes for methicillin resistance, which has been found to complicate mastitis treatment with β-lactam antibiotics [32].

PFGE analysis was conducted to identify the clonal identity and genetic relatedness of the recovered *S. aureus* isolates, with a particular focus on the source of the samples. According to the results of the dendrogram analysis (Figure 2), the phylogenetic analysis revealed close genetic relatedness among the ovine-recovered strains. The similarity of indices among ovine mastitis strains recovered from the same farm was significant (similar indices of over 90%; p-value 0.05). Furthermore, strains collected from different farms showed >80% genetic relatedness. A common source of clonality exists, with a dominant clone among *S. aureus* isolated from ovine mastitis. Our study revealed that genetically related clones of *S. aureus* exhibit distinct behaviors. In general, these clones tend to cause mastitis in ovines with low penetration and transmission to humans and hospitals. The PFGE analysis revealed no correlation between *S. aureus* isolates originating from ovine or human sources from the same farm or region. Although numerous studies have documented genetic relatedness between *S. aureus* isolates from farm animals and those in close contact with them, other studies have not identified any significant relationship between these isolates. According to a study conducted in Tanzania comparing *S. aureus* isolates from raw cows’ milk and humans working in the same dairy farm, distinct sequence types were found, indicating that interspecies transmission is rare [34]. In contrast, research conducted in the Netherlands on methicillin-susceptible *S. aureus* carriage isolates from various farm animals and humans revealed high genetic diversity and no clear evidence of direct transmission [35]. The findings of this study highlight the complexity of the epidemiology of *S. aureus*. Although zoonotic and reverse zoonotic transmission is possible, it is not universally observed and depends on numerous factors, such as strain characteristics, environmental conditions, and biosecurity measures. In addition to PFGE, *spa* typing is a rapid and accurate method of distinguishing outbreak isolates from local and global epidemiological studies, with the ability to transfer sequence data between laboratories over the Internet [7]. *spa* typing of *S. aureus* isolates from ovine milk provides valuable insight into the dissemination patterns of strains associated with clinical and subclinical mastitis in sheep [36]. According to the results of this study, *spa* type t1534 was the most prevalent type, detecting 81.25% of ovine *S. aureus* isolates (n = 13). *Spa* type t1534 is associated with ovine mastitis in several countries, including Algeria and England, at different frequencies [1, 37]. Furthermore, *spa* t1534 was isolated from the nares of healthy goats and sheep. Based on these findings, the nares of these animals could serve as a natural reservoir for this pathogen as well as a source of contamination for the udders and milk of ovines [4, 38]. Our remaining *S. aureus* isolates associated with ovine contained *spa* types t1736 and t845.

*Spa* type t1736 was found in *S. aureus* isolates from bovine mastitis [39], human clinical specimens [40], and wild rodents [41]. However, the third *spa* type identified in this study (t845) has only been reported in human clinical samples [41, 42]. The dendrogram clearly distinguishes between the *spa* types associated with *S. aureus* recovered from animal handlers, separating those associated with humans from those associated with ovines. This may indicate that cross-transmission of *S. aureus* strains between sheep and animal handlers is uncommon (Figure 2). *spa* type t223 was detected in 74% (n = 3) of our human *S. aureus* isolates, and it is one of the most reported *spa* types in Jordan; it has been recovered from healthy adults and children in the community, highly touched surfaces, and human clinical specimens [43–45]. This study found that the fourth human-associated isolate was *spa* type t304, which was found in bovine subclinical mastitis, raw milk cheese samples, and nasal swabs of food workers [46, 47].

## CONCLUSION

This study revealed that *S. aureus spa* type t1534 is a prominent cause of ovine mastitis in Jordan. In addition, the results of the phylogenetic analysis of the strains recovered after PFGE revealed that the strains were closely genetically related. This was coupled with a low level of penetration and transmission of the isolates from ovine herds to animal handlers working on ovine farms. In addition, several ARGs were detected in the ovine isolates. 12.5% (n = 2) of the isolates contained *mecA*. This is particularly relevant because *mecA* encodes methicillin resistance, which makes treating mastitis with β-lactam antibiotics challenging. In light of these findings, further research should be conducted on the relationship between *S. aureus* strains associated with mastitis and those associated with human disease, especially in the context of a One-Health strategy that incorporates continuous surveillance.

## AUTHORS’ CONTRIBUTIONS

MHG: Conceptualization, methodology, investigation, formal analysis, and drafted, reviewed, and edited the manuscript. LFA: Methodology, formal analysis, and drafted the manuscript. TAM: Methodology, investigation, data curation, and drafted the manuscript. MK: Formal analysis and drafted, reviewed, and edited the manuscript. AA: Methodology, formal analysis, and reviewed and edited the manuscript. All authors have read, reviewed, and approved the final manuscript.
